# Calling in the Ca_V_alry—*Toxoplasma gondii* Hijacks GABAergic Signaling and Voltage-Dependent Calcium Channel Signaling for *Trojan horse*-Mediated Dissemination

**DOI:** 10.3389/fcimb.2019.00061

**Published:** 2019-03-20

**Authors:** Amol K. Bhandage, Antonio Barragan

**Affiliations:** Department of Molecular Biosciences, The Wenner-Gren Institute (MBW), Stockholm University, Stockholm, Sweden

**Keywords:** apicomplexa, CNS infection, dendritic cell, microglia, motility, GABA receptor

## Abstract

Dendritic cells (DCs) are regarded as the gatekeepers of the immune system but can also mediate systemic dissemination of the obligate intracellular parasite *Toxoplasma gondii*. Here, we review the current knowledge on how *T. gondii* hijacks the migratory machinery of DCs and microglia. Shortly after active invasion by the parasite, infected cells synthesize and secrete the neurotransmitter γ-aminobutyric acid (GABA) and activate GABA-A receptors, which sets on a hypermigratory phenotype in parasitized DCs *in vitro* and *in vivo*. The signaling molecule calcium plays a central role for this migratory activation as signal transduction following GABAergic activation is mediated via the L-type voltage-dependent calcium channel (L-VDCC) subtype Ca_v_1.3. These studies have revealed that DCs possess a GABA/L-VDCC/Ca_v_1.3 motogenic signaling axis that triggers migratory activation upon *T. gondii* infection. Moreover, GABAergic migration can cooperate with chemotactic responses. Additionally, the parasite-derived protein Tg14-3-3 has been associated with hypermigration of DCs and microglia. We discuss the interference of *T. gondii* infection with host cell signaling pathways that regulate migration. Altogether, *T. gondii* hijacks non-canonical signaling pathways in infected immune cells to modulate their migratory properties, and thereby promote its own dissemination.

## Introduction

The apicomplexan parasite *Toxoplasma gondii* infects a diverse repertoire of hosts, including humans and rodents (Tenter et al., 2000). As an obligate intracellular pathogen, *Toxoplasma* is able to infect and replicate within virtually any type of nucleated cell from warm-blooded vertebrates. Despite that up to one-third of humans become chronically infected during their lifetime, most infections are considered asymptomatic. Yet, *Toxoplasma* can cause life-threatening disease in immunocompromised individuals or to the developing fetus (Montoya and Liesenfeld, 2004). Congenital toxoplasmosis is also a significant problem in veterinary medicine.

After oral ingestion, systemic parasite dissemination during primary infection precedes the establishment of chronic infection. Early studies in rodents showed that, shortly after invasion of the intestinal tissue (Dubey, 1997), parasites (tachyzoite stage) are retrieved in the blood circulation (Derouin and Garin, 1991; Zenner et al., 1998). At this early stage–before the infection is controlled by the immune response–the parasite can be isolated from virtually any organ in rodents, including immunoprivileged organs such as brain, eyes, and testis (Hitziger et al., 2005). Consequently, breaching restrictive biological barriers, such as the intestine, the blood brain-barrier, blood-retina barrier, or the placenta, is a requisite for the establishment of primary *Toxoplasma* infection and subsequently chronic infection or congenital infection.

The rapidly replicating stage of *T. gondii* (the tachyzoite) mediates dissemination. Tachyzoites have an obligate intracellular existence and their active locomotion, termed gliding motility, is instrumental for invasion of host cells (Dobrowolski and Sibley, 1996). Gliding motility also powers parasite migration within the tissue microenvironment (Barragan and Sibley, 2002). Additionally, transportation by the blood and the lymphatic circulation assures rapid systemic dissemination to distant organs.

The onset of immune responses against *T. gondii* is accompanied by the transformation of the parasite into tissue cysts (bradyzoite stage) and is believed to result in long-lasting or lifelong chronic infection in humans (Joynson and Wreghitt, 2001). In rodents, cellular immune responses mediated by dendritic cells (DCs), T cells, NK cells, macrophages, and cytokine responses (IL-12 and IFN-γ) are essential to overcome primary infection and for establishing latent chronic infection (Yap and Sher, 1999; Sacks and Sher, 2002). Leukocytes traffic the tissues, populate the blood, drain to the lymphatic system and back into the circulation (Friedl and Weigelin, 2008). Thus, while constitutive epithelial and connective tissues may provide refuge and replicative niches for *T. gondii*, leukocytes additionally mediate immune surveillance and are essential for pathogen clearance. However, the migratory functions of leukocytes make them also suitable vehicles for *Toxoplasma* to mediate its dissemination in the organism by a *Trojan horse* mechanism (Weidner and Barragan, 2014).

In this review, we discuss the current knowledge on how *Toxoplasma* modulates the migratory properties of immune cells, primarily DCs, and microglia, by inducing a hypermigratory phenotype that promotes parasite dissemination. The mechanisms involved in *Toxoplasma*-induced hypermigration include a hijacking of the GABAergic signaling system and voltage-dependent calcium channel (VDCC) signaling in parasitized cells.

## GABA, a Signaling Molecule in the CNS and Peripheral Tissues

The γ-aminobutyric acid (GABA) is most commonly known as an inhibitory neurotransmitter in the CNS of vertebrates, where it contributes in maintaining the balance between excitatory and inhibitory neurotransmission. GABA executes its action in the CNS through GABA-A and GABA-B receptors. GABA-A receptors are chloride (Cl^−^) -permeable ion channels formed by pentameric combinations of subunits (2 α + 2 β + 1 additional subunit) out of 19 known subunits to date (α1-6, β1-3 γ1-3, δ, ε, θ, π, ρ1-3) (Olsen and Sieghart, 2008; Sieghart et al., 2012). In contrast, GABA-B receptors are metabotropic G-protein coupled receptors formed as a heterodimer of 2 subunits (Bowery et al., 2002; Bowery, 2010).

The depolarizing (excitatory) or hyperpolarizing (inhibitory) effects of GABA depend on the intracellular Cl^−^ concentration [Cl^−^]_i_ which is set by cation chloride co-transporters (CCCs) -NKCCs and KCCs (Blaesse et al., 2009; Kaila et al., 2014). For instance, higher relative expression of NKCC1 than KCC2 in the immature neurons results in higher [Cl^−^]_i_ leading to depolarizing effects of GABA (Ben-Ari et al., 2007; Kilb, 2012). Thus, GABA can mediate depolarizing or hyperpolarizing responses in immature and mature neurons, respectively, indicating a developmental switch between the actions of GABA. Additionally, certain neuronal populations in the adult rat brain exhibit depolarizing responses to GABA (Chiang et al., 2012; Haam et al., 2012; Sauer et al., 2012). Further, experimental down-regulation of KCC2 results in excitatory effects upon GABA-A receptor activation in the adult neurons (Sarkar et al., 2011). Overall, these studies highlight that the actions of GABA are highly dependent on activity of Cl^−^ transporters, developmental stage, cell type and sub-cellular localization (Bortone and Polleux, 2009).

Beyond neurotransmission, GABA has also been attributed roles in cellular processes such as migration, proliferation, differentiation, synapse formation, axonal growth, and neuronal death (Birnir and Korpi, 2007; Kilb, 2012). In addition to its predominant presence in the CNS, GABA is also synthesized by non-neuronal tissues like pancreatic islets, glia cells, adrenal medulla, germ cells, testes, and immune cells and these tissues also often express GABA receptors (Gladkevich et al., 2006; Jin et al., 2013). Of note, GABA precedes the existence of the vertebrate and invertebrate nervous systems and is synthesized by prokaryotes for metabolic purposes (Feehily et al., 2013; Xiong et al., 2014), and by plants for signaling (Michaeli and Fromm, 2015). *T. gondii* possesses a GABA shunt pathway and can utilize GABA as an energy source, for instance, to sustain gliding motility under nutrient-limited conditions (MacRae et al., 2012). Thus, GABA's function as a neurotransmitter is likely an evolutionary adaption posterior to metabolic and other signaling functions. In other words, GABA precedes the development of a CNS in metazoa and, consequently, alternative functions exist.

## A GABAergic Signaling System in Immune Cells and Peripheral Tissues

Research over the past decade has identified the functional implications of GABA and its receptors outside the CNS, i.e., in pancreatic islets, immune system, digestive system, and reproductive system (Gladkevich et al., 2006; Bhandage et al., 2018a; Korol et al., 2018). Mounting evidence indicates that brain-resident immune cells, such as microglia, and circulating immune cells, such as neutrophils, T lymphocytes, monocytes, macrophages and DCs, secrete GABA, and/or express GABA receptors and associated proteins (Rane et al., 2005; Alam et al., 2006; Bhat et al., 2010; Lee et al., 2011; Fuks et al., 2012; Bhandage et al., 2015, 2018a).

Further, GABA impacts on the effector functions of immune cells, i.e., migration, proliferation, and cytokine secretion via GABA receptors. For instance, GABA mediates protective roles by inhibiting auto-reactive T cells in human autoimmune inflammatory diseases such as type 1 diabetes, rheumatoid arthritis and multiple sclerosis (Tian et al., 2004, 2011; Bhat et al., 2010; Bhandage et al., 2018a). Gephyrin-dependent enhanced GABA signaling has been shown to participate in the conversion of alpha cells to insulin producing beta cells in diabetic pancreatic islets resulting in cure of type 1 diabetes (Li et al., 2017). GABA and GABA-A receptor signaling have also been implicated in maturation and proliferation of adult stem cells and GABA has been suggested as a tumor-signaling molecule in cancer cells (Andäng et al., 2008; Young and Bordey, 2009). Moreover, a recent study demonstrates antimicrobial and autophagy-related roles for GABA in macrophages upon mycobacterium infection (Kim et al., 2018). Jointly, this highlights the significance of GABAergic signaling in human diseases.

Thus, mounting evidence shows that the immune system harbors components or the complete machinery for GABAergic signaling and that GABA can serve as a modulator of the effector functions of immune cells (Table 1). It evidences a possible cross-talk between the nervous system and the immune system and the presence of novel “neuro-immune-signaling” axes (Sospedra and Martin, 2005; Levite, 2012). However, the understanding of GABAergic signaling and its patho-/ physiological roles in immune cells is still rudimentary or non-existent compared with neuronal systems and thus, crucial to be further studied in depth.

**Table 1:** GABAergic and VDCC components described in cells of the immune system.

**Acronym**	**Full name**	**Function**	**Expressing cells**	**Species**	**References**
GAD	Glutamate decarboxylase	GABA synthesis	DC, T cell, microglia	Human, mouse	Dionisio et al., 2011; Fuks et al., 2012; Bhandage et al., 2019
GABA-T	GABA-transaminase	GABA degradation	T cell, microglia	Human, mouse	Dionisio et al., 2011; Bhandage et al., 2019
GAT	GABA transporter	GABA transportation across the cell membrane for secretion	DC, microglia, T cell	Human, mouse	Dionisio et al., 2011; Fuks et al., 2012; Bhandage et al., 2019
GABA-A R	GABA-A receptors	Polarization/depolarization of the cell membrane via Cl^−^ flux into/out of the cell	DC, PBMC^a^, T cell, monocyte, macrophage, microglia	Human, mouse, rat	Tian et al., 2004; Bhat et al., 2010; Dionisio et al., 2011; Wheeler et al., 2011; Fuks et al., 2012; Mendu et al., 2012; Bhandage et al., 2015, 2018a, 2019
NKCC	Na^+^-K^+^-Cl^−^ co-transporter	GABA-A R regulation via Cl^−^ transport into the cell	PBMC, microglia	Human, mouse	Bhandage et al., 2015, 2018a, 2019
KCC	K^+^-Cl^−^ co-transporter	GABA-A R regulation via Cl^−^ transport out of the cell	PBMC, microglia	Human, mouse	Bhandage et al., 2015, 2018a, 2019
VDCC (Ca_V_)	Voltage-dependent calcium channels	Ca^2+^ signaling via flux into/out of the cell	DC, PBMC, microglia	Human, mouse	Kanatani et al., 2017; Bhandage et al., 2018b, 2019

a *peripheral blood mononuclear cells*.

## VDCC Signaling in Immune Cells

VDCCs are formed from α1, α2, β, δ, and γ subunits where the transmembrane α1 subunit forms the tetrameric ion channel with a central pore permeable specifically for calcium ions. The 10 members of the VDCC (also called Ca_V_) family can be divided in 3 subfamilies—high voltage activated L-type, moderate voltage activated P/Q, N, R-type and low voltage activated T-type channels (Catterall, 2011).

The expression of VDCCs by immune cells has long remained unresolved. However, their expression and implication in immune functions has more recently received attention (Table 1). In excitable cells like neurons and pancreatic islets beta cells, the channels are opened by depolarizing voltage changes in the membrane potential but how they are operated in immune cells is still ambiguous (Badou et al., 2013). It is possible that immune cells can sense these depolarizing changes by activation of ligand-gated ion channels such as GABA-A receptors and N-methyl-D-aspartate (NMDA) receptors.

Some of the implications of VDCCs in the immune cell physiology are described here. The Ca_V_1.4 channels, L-type VDCC, have been shown to contribute in controlling naive T cell homeostasis, T cell receptor (TCR) signaling and antigen-driven T cell immune responses (Omilusik et al., 2011). Additionally, Ca_V_1.2 and Ca_V_1.3, other L-type VDCC channels, participate in TCR-induced calcium flux in T cells (Stokes et al., 2004). Not only α subunits but also β subunits of Ca_V_ channels are shown to be important for normal T cell functions such TCR-mediated calcium entry, nuclear factor of activated T cells (NFAT) activation, and cytokine production (Badou et al., 2006). The Ca_V_3.1, T-type channel, shapes the cytokine profile of T helper cells and can ameliorate autoimmune responses (Wang et al., 2016). The Ca_V_1.2 channels can activate intracellular calcium receptors and contribute in the surface expression of MHC class II molecules in DCs during antigen presentation to T cells (Vukcevic et al., 2008). While characterization has just begun, these evidences indicate functional roles for VDCCs in immune cell physiology.

## Infection of Leukocytes By *T. gondii* and Their Role in Parasite Dissemination

Following oral infection, *Toxoplasma* rapidly disseminates in its host. Early studies in rodents detected tachyzoites in the blood, lymph nodes and peripheral organs rapidly after infection (Derouin and Garin, 1991; Dubey, 1997; Zenner et al., 1998). It was demonstrated that not only the intestinal tissue becomes parasitized but also the intra-epithelial leukocytes (Dubey et al., 1997). Following studies showed that both resident and non-resident leukocytes become infected in the intestine (Courret et al., 2006; Gregg et al., 2013).

**Figure 1:**
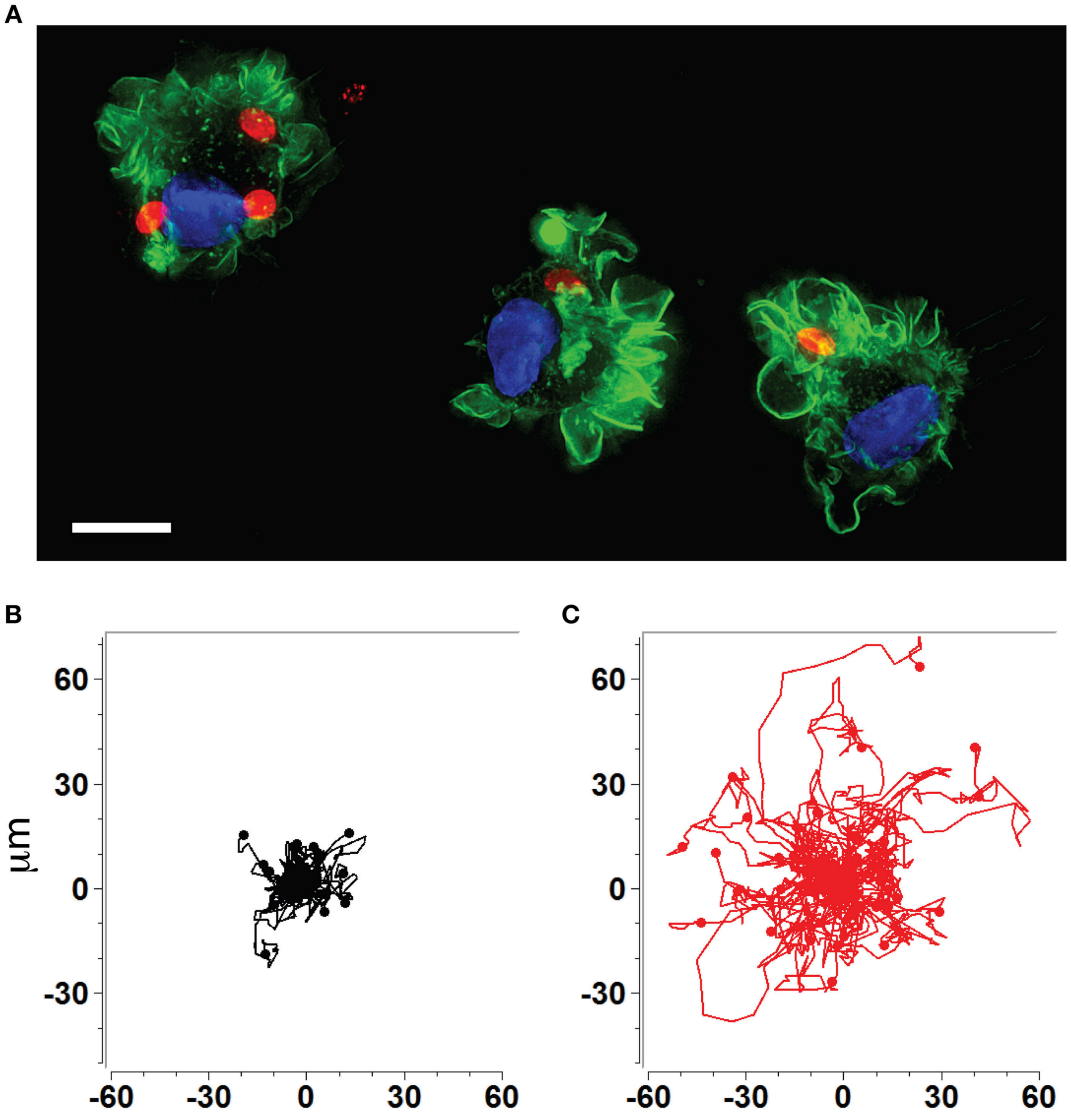
Human and murine DCs exhibit a hypermigratory phenotype upon infection by *T. gondii*. **(A)** Human monocyte-derived DCs (hMDDCs) infected with RFP-expressing *T. gondii* tachyzoites (red) stained with Alexa flour 488 Phalloidin to detect F-actin (green). Cell nuclei are stained with DAPI (blue). Shortly after tachyzoite invasion, cells undergo rapid morphological changes, including rounding-up, accentuation of membrane projections such as veils and ruffles, and dissolution of podosomes. Scale bar = 10 μM. **(B,C)** Representative motility plots of unchallenged (black) and *Toxoplasma*-infected (red) murine bone marrow-derived DCs (mBMDCs), respectively. Infected cells exhibit prolonged migratory paths and elevated velocities. Cells were imaged and tracked as described in Weidner et al. (2013). X- and Y-axes indicate μm.

The rapid dissemination of *T. gondii* tachyzoites (Hitziger et al., 2005) combined with the parasite's ability to infect and replicate within leukocytes (Channon et al., 2000) raised the hypothesis that the systemic spread of parasites was mediated by a *Trojan horse* type of mechanism. DCs were identified as important mediators of dissemination and attributed shuttling functions for *T. gondii* (Courret et al., 2006; Lambert et al., 2006; Bierly et al., 2008). Additionally, all strains tested to date from the three predominant *T. gondii* lineages (types I, II, III) induce a hypermigratory phenotype in DCs upon challenge with tachyzoites (Figures 1A–C) (Lambert et al., 2009). The characteristics and criteria of the hypermigratory phenotype have been previously reviewed (Weidner and Barragan, 2014) and its impact on the dissemination of *T. gondii* is discussed below.

Similar to DCs, monocytic cells have been attributed a hypermigratory phenotype upon *Toxoplasma* challenge (Harker et al., 2013; Cook et al., 2018). Additionally, parasite-transportation functions to the brain have been attributed to monocytes (Courret et al., 2006; Lachenmaier et al., 2011). Other leukocytes also become infected *in vivo* and thus may also contribute to the systemic spread of the infection. These include T cells (Persson et al., 2007; Chtanova et al., 2009), NK cells (Persson et al., 2009; Sultana et al., 2017), neutrophils (Norose et al., 2008; Coombes et al., 2013), and macrophages (Da Gama et al., 2004; Lambert et al., 2011). While NK cells do not seem to facilitate passage of *T. gondii* across the blood-brain barrier (Petit-Jentreau et al., 2018), the relative contribution of the different leukocyte types to dissemination at the different phases of the infection remains undetermined.

## *T. gondii* Infection and GABAergic Signaling in DCs

### Primary Human and Murine DCs Express a Functional GABAergic System

**Figure 2:**
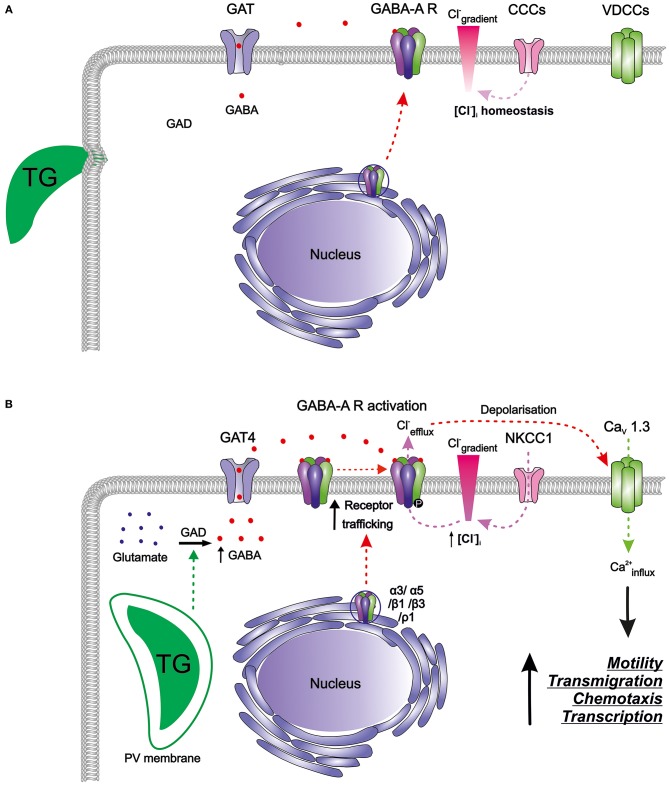
The GABAergic motogenic signaling axis that mediates the migratory activation of DCs and microglia upon *T. gondii* (TG) infection. **(A)** Resting DCs and microglia harbor components of the GABAergic machinery including enzymes for GABA synthesis (GAD), GABA transporters (GATs), GABA-A receptors (GABA-A R), chloride co-transporters (CCCs), and also the components of the VDCC signaling system, with calcium as a second messenger. GABA is produced from glutamate by glutamate decarboxylases (GAD65/67) and is transported by GATs. GABA-A R subunits are transcribed and assembled in the ER-Golgi compartment before trafficking to the plasma membrane. Activation of GABA-A R requires binding of two GABA molecules to open the ion channel pore permeable for chloride (Cl^−^). The CCCs i.e., NKCCs/KCCs are involved in the maintenance of Cl^−^ gradient in DCs. In murine DCs, expression of the L-type VDCC Ca_V_1.3 predominates over other expressed VDCCs while microglia express a broader set of VDCCs. **(B)** *T. gondii* actively invades host cells and resides intracellularly in a parasitophorous vacuole (PV). Shortly after parasite invasion, DCs exhibit (i) enhanced GABA synthesis through GAD, (ii) upregulation of the transporter GAT4, (iii) elevated expression of GABA-A R subunit mRNAs, indicative of increased receptor trafficking to the membrane, and (iv) elevated NKCC1 activity leading to an increase in [Cl^−^]_i_. A similar GABAergic activation occurs in microglia upon *Toxoplasma* infection. The secreted GABA acts in an autocrine fashion and activates GABA-A R. Opening of GABA-A channels results in Cl^−^ efflux from the cell producing depolarization of the plasma membrane. Depolarization activates VDCCs, preferentially the subtype Ca_V_1.3 in DCs, and leads to calcium (Ca^2+^) entry into the cell. Hypothetically, Ca^2+^ acts as second messenger to promote cellular signaling implicated in motility, transmigration, chemotaxis and transcriptional modulation. Hitherto unidentified intracellular targets may include 14-3-3-regulated MAP kinase activity. Higher intracellular Ca^2+^ concentrations or fluxes are likely required for the observed rapid cytoskeletal rearrangements, such as the dissolution of podosomes and integrin redistributions implicated in the hypermigratory phenotype.

Murine bone marrow-derived DCs (mBMDCs) express mRNAs for five GABA-A receptor subunits (α3, α5, β1, β3, ρ1), the enzyme responsible for GABA synthesis (glutamate decarboxylase GAD65) as well as a GABA transporter (GAT4) (Fuks et al., 2012). Since the classical GABA-A channel pentamer requires 2 α, 2 β, and 1 additional subunit, the expression pattern in mBMDCs indicated the possibility of subunit assembly into a pentamer that can be trafficked to the cell membrane (Figure 2A). GABA-induced whole-cell inwards currents recorded in mBMDCs and human monocyte-derived DCs (hMDDCs) using patch clamp electrophysiology (Fuks et al., 2012) demonstrated presence of functionally active GABA-A receptors. It may be assumed that the activation of GABA-A receptors results in efflux of Cl^−^ ions out of cells, leading to membrane depolarization. Thus, DCs harbor a functional GABAergic system.

Knowledge remains limited on the expression and function of the GABAergic system in other immune cells (Barragan et al., 2015). It will be instrumental to determine the subunit composition of the active subtype of GABA-A receptors and of additional GABAergic signaling components such as CCCs, GADs, GATs, GABA transaminase (GABA-T), and GABA-A receptor anchoring proteins. Further extending this knowledge to human DCs (native myeloid/plasmacytoid DCs) will be crucial to understand the implication of GABAergic signaling in human diseases.

### *Toxoplasma*-Infected DCs Secrete GABA

Independent of the parasite strain, *Toxoplasma* infection induced GABA secretion in mBMDCs in a time- and dose-dependent manner (Fuks et al., 2012). Further, hMDDCs also consistently secreted GABA upon parasite infection with a donor-to-donor quantitative variability (Fuks et al., 2012). Parasitized DCs, but not by-stander DCs, secreted GABA, indicating that intracellular localization of live parasites was necessary for GABAergic activation of DCs. In line, GABA secretion was associated with active tachyzoite invasion but not adhesion of parasites to the cell membrane, challenge with heat-inactivated tachyzoites or lysate, infected cell supernatants and LPS. Pharmacological inhibition of GABA synthesis or transport did not impact on parasite replication inside DCs or on the DC viability, suggesting that GABAergic signaling was independent of the host cell and parasite developments.

GABAergic cells are defined by their ability to produce GABA through the expression of GABA synthesizing enzymes. GAD has two isoforms: GAD65 and GAD67 (Kilb, 2012). The abundant secretion of GABA and expression of GAD65 in *Toxoplasma* infected-DCs show that DCs become GABAergic cells upon parasite infection.

It remains unknown how the parasite infection regulates GABA synthesis and secretion by DCs. It is plausible that parasite effector molecules interact with components of the GABAergic system such as enzymes synthesizing or metabolizing GABA (GADs) and GABA-T or transporters of GABA (GATs) to induce these secretions (Figure 2B). Upon parasitic infection in mBMDCs, transcriptional expression of GAD65 was unaltered but expression of GAT4 was seven-fold upregulated (Fuks et al., 2012). Also, the intervention in GABA synthesis by pharmacological inhibition of GAD (using semicarbazide) abolished GABA secretion in the supernatants whereas inhibition of the transporter GAT4 (using SNAP 5114) diminished GABA secretion to around half (Fuks et al., 2012; Kanatani et al., 2017). This suggests that GABA is not only synthesized *de novo*, but also transported more efficiently outside the cells implying roles for both GAD65 and GAT4 in the hypermigratory phenotype.

Of note, *Toxoplasma* utilizes a metabolic GABA-shunt as an energy source under nutrient-limited conditions (MacRae et al., 2012). In respect to hypermigration of parasitized DCs, inhibition of GABA synthesis or transport decreased the concentration of GABA only in the supernatants of infected DCs but not in the supernatants from free (extracellular) tachyzoites, indicating that the host cell GABAergic machinery is responsible for the augmented secretions of GABA.

Recently (Brooks et al., 2015) identified delocalization of GAD67 in *Toxoplasma*-infected rodent brain as an indication of disturbed GABA signaling in CA3 pyramidal neurons and increased susceptibility of the animals to experimentally induced epileptic seizures. If GAD67 plays a role in the hypermigration of primary DCs remains to be investigated.

### Modulation of GABA-A Receptors in Parasitized DCs

The abundant GABA secretions in the supernatants of infected DCs would presumably act in an autocrine fashion on GABA-A receptors in the host cell membrane. Since GABA-A receptors are fast Cl^−^ ion channels with opening time in the millisecond range and [Cl^−^]_i_ in monocytic cells is higher than in mature neurons (ranging from 24 to 75 mM), GABA-mediated activation of GABA-A channels in DCs results in inwards currents, as shown by patch clamp electrophysiology (Fuks et al., 2012) i.e., efflux Cl^−^ ion out of the cell. Thus, GABAergic activation will increase the positive charge inside the cell and depolarize the plasma membrane (Tian et al., 2004; Cahalan and Chandy, 2009; Bhandage et al., 2018a).

As indicated above, mBMDCs express mRNAs for five GABA-A receptor subunits (α3, α5, β1, β3, ρ1). Interestingly, the expression of α3 and ρ1 subunits was upregulated by 2 h post-infection (Fuks et al., 2012). This probably contributes to *de novo* assembly of GABA-A pentamers that can traffic to the membrane. Increase in the number of functional GABA-A pentamers on the membrane would enhance GABAergic signaling, and thereby depolarization in DCs (Figure 2B). GABA has been shown to induce migration and chemotaxis in immature neurons *in vitro* through the activity of ρ subunit-containing GABA-A receptors (Denter et al., 2010). Similarly, the upregulation of the ρ1 subunit in *Toxoplasma*-infected DCs raises the possibility that ρ1-containing GABA-A receptors may be involved in the hypermigration of DCs. Further, parasitic infection may possibly impact on intracellular molecules such as protein kinases that regulate GABA-A receptor activity. Given the expression level of GABA-A receptor subunits and their functional status upon parasite infection in mBMDCs, we speculate that opening of few GABA-A channels per cell per unit time may be sufficient to change the membrane potential significantly, further activating downstream signaling (Chandy et al., 2004; Tian et al., 2004; Cahalan and Chandy, 2009; Feske et al., 2012).

Altogether, *Toxoplasma*-induced activation of GABAergic signaling will depolarize the DCs, reaching the threshold membrane potential for opening of VDCCs and resulting in calcium entry into the cell. Previous reports have shown that depolarizing GABA-A receptor activation can cause calcium influx through VDCCs without triggering action potentials in immature neurons and stimulate neuronal migration, chemotaxis and maturation (Lin et al., 1994; Behar et al., 1996; Ganguly et al., 2001; Owens and Kriegstein, 2002; Ben-Ari, 2012). It was therefore reasonable to explore whether *T. gondii* infection induced DC migration through a similar mechanism, as delineated below.

### GABAergic Signaling Mediates Enhanced Transmigration and Hypermotility of *Toxoplasma*-Infected DCs

GABA-A receptor blockade inhibits hypermigration of *Toxoplasma*-infected DCs (Fuks et al., 2012). Similarly, interference of GABA synthesis or transportation by pharmacological inhibition of GADs or GATs, respectively, abrogates transmigration and hypermotility in parasitized DCs. Addition of supernatants from infected DCs or exogenous GABA in presence of the inhibitors of GAD and GAT reconstituted the transmigration frequencies and hypermotility of DCs (Kanatani et al., 2017). Further, adoptively transferred parasitized DCs in mice exhibited impaired migration and reduced dissemination upon treatment with inhibitors of GAD and GAT as compared to non-treated cells. Additionally, the parasite load in brain, spleen and mesenteric lymph nodes of mice was substantially lower upon GABAergic inhibition. This suggests that the hypermigratory phenotype cannot be induced without active GABAergic signaling in DCs.

### Regulation of the GABA-A Receptor Activity by CCCs

The activity of GABA-A receptors i.e., depolarization (excitation) or hyperpolarization (inhibition) depends on the [Cl^−^]_i_ which, in turn, is set by CCCs. The most commonly studied CCCs in the CNS are NKCC1 and KCC2 out of 2 and 4 from NKCC and KCC family of solute carriers, respectively, and their expression varies depending on developmental stages, cell types and sub-cellular localization (Blaesse et al., 2009; Kaila et al., 2014). Thus, CCCs are essential for maintaining Cl^−^ homeostasis to obtain depolarizing or hyperpolarizing GABAergic responses (Glykys et al., 2014).

Since *Toxoplasma* infection modulates the GABAergic system in DCs, it would be interesting to know which CCCs are instrumental in DCs for maintenance of [Cl^−^]_i_. Any changes in CCC expression could alter [Cl^−^]_i_ which eventually will alter the activity of functional GABA-A receptors in DCs. Some evidences indicate that monocytic cells have significantly higher [Cl^−^]_i_ than that of mature neurons (Ince et al., 1987; DeFazio et al., 2000). With this high [Cl^−^]_i_, DCs ought to depolarize upon GABA-A receptor activation, similar to the depolarizing effects of GABA on immature neurons (Kilb, 2012). However, the CCC expression repertoire of CCC in DCs and the putative impact by *Toxoplasma* infection remain uncharacterized. Yet, recent data indicates that the CCC expression is modulated in primary microglia upon *Toxoplasma* infection (Bhandage et al., 2019).

## Calcium Signaling Downstream to GABAergic Signaling Is Essential for the Induction of a Hypermigratory Phenotype in DCs

### Ca_**V**_1.3 Channels Are the Active L-Type VDCCs Mediating Hypermigration in DCs

In sub-physiological concentrations of calcium, the *Toxoplasma*-induced hypermigratory phenotype of DCs was abrogated (Kanatani et al., 2017). In addition, nickel ions dose-dependently inhibited induction of hypermotility. Since nickel ions compete with physiological calcium by blocking the permeation path of VDCCs, this suggested the involvement of VDCCs.

A transcriptional analysis showed that mBMDCs constitutively expressed 9 different Ca_V_ channel pore-forming α1 subunits, indicating a possibility for formation of functional Ca_V_ channels (Kanatani et al., 2017). When the most prominently expressed Ca_V_1.3 channels were pharmacologically inhibited or genetically silenced, the abolished transmigration and hypermotility in mBMDCs were not recovered by addition of exogenous GABA (Figure 2B). Inhibition of other calcium channels, e.g., silencing of Ca_V_1.2 channels or antagonism of purinergic receptors, non-significantly impacted on hypermotility, indicating that Ca_V_1.3 was the key VDCC subtype in DCs responsible for mediating *Toxoplasma*-induced hypermigration.

Additionally, calcium influx through L-type VDCCs can regulate the activity of GABA-A receptors through phosphorylation of the β3 subunit by calmodulin-dependent protein kinase II (CaMKII) (Saliba et al., 2012). This suggests that Ca_V_1.3 channels and GABA-A receptors may cooperate and mutually regulate each other in DCs.

### GABAergic Signaling Induces VDCC-Mediated Calcium Signaling in DCs

Exposure of DCs to GABA evoked a transient calcium influx, suggesting that GABA-A receptor-induced depolarization can open VDCCs for calcium influx (Kanatani et al., 2017). Additionally, inhibition of GABAergic signaling abrogated hypermotility, which was reconstituted by VDCC activation (Bay-K8644). In sharp contrast, GABAergic activation was unable to recover the hypermotility of DCs upon blockade of Ca_V_ channels by broad inhibitors of VDCCs (nifedipine, benidipine), by a selective Ca_V_1.3 channel antagonist or by Ca_V_1.3 gene silencing. Jointly, this indicated that VDCC-dependent calcium signaling mediated hypermotility downstream of GABAergic signaling (Figure 2B).

### Calcium Signaling Through VDCCs Results in Hypermigration

Calcium, being an important second messenger, participates in multiple cellular processes. Local openings of VDCCs on the plasma membrane are crucial for neurotransmission in neurons or the fusion of insulin-containing vesicles in pancreatic islet beta cells (Lin et al., 1994; Behar et al., 1996; Gandasi et al., 2017). Similarly, in *Toxoplasma*-infected hypermotile DCs, the subcellular microdomains in the plasma membrane may be important for signaling, and therefore for conferring the migratory activation. The observed transient calcium fluxes in response to GABA may participate in the dissolution of adhesion-mediating podosomes (Weidner et al., 2013), the redistribution of integrins (Kanatani et al., 2015), the balance between activity of matrix metalloproteinases (MMPs) and tissue inhibitor of metalloproteinase 1 (TIMP1) (Olafsson et al., 2018) and thus, ultimately in the cytoskeletal remodeling driving the DCs toward a hypermigratory phenotype. Of note, TIMP1 is released by calcium-dependent vesicular exocytosis (Dranoff et al., 2013) and is instrumental in the reduced proteolytic activity and migratory activation of parasitized DCs (Olafsson et al., 2018). Possibly, high-resolution live cell calcium imaging might elucidate the regulation of these processes by calcium.

Why is *Toxoplasma* manipulating inotropic receptors in the host cell? The effector functions achieved through the modulation of inotropic receptors are rapid, in range of seconds, and therefore offer the advantage of bypassing transcriptional regulation in the host cell. Thus, it might be in favor of *Toxoplasma* to hijack GABAergic and VDCC signaling in DCs to achieve a rapid onset of hypermigration shortly after host-cell invasion and thereby speed-up the process of systemic dissemination (Lambert et al., 2006; Fuks et al., 2012).

## Modulation of Ionotropic Signaling in Microglia and Other Brain-Resident Cells

Recent work has shown that *Toxoplasma* infection can alter GABAergic synapses in the rodent brain (Brooks et al., 2015). Altering of the distribution of the GABA synthesis enzyme GAD67 led to disturbed GABA signaling and increased the susceptibility of animals to experimentally induced epileptic seizures. Also, *Toxoplasma* infection decreased the expression of the astrocytic glutamate transporter, GLT1, and increased extracellular glutamate levels (David et al., 2016). Thus, the observed dysregulation of GABAergic and glutamatergic signaling in toxoplasmosis is intriguing, also in perspective of the infiltration of DCs to the brain parenchyma during *Toxoplasma* infection (John et al., 2011). Additionally, astrocytes and microglia are both permissive to parasite invasion and replication *in vitro* but only microglia exhibited enhanced transmigration and hypermotility upon challenge with *T. gondii* (Dellacasa-Lindberg et al., 2011; Contreras-Ochoa et al., 2012; Bhandage et al., 2019). This raises the question whether microglia serve as *Trojan horses* for *Toxoplasma* dissemination within the brain parenchyma.

Human microglia have been reported to express GABA-T and 3 GABA-A receptor subunits (α1, α3, and β1) (Lee et al., 2011). In addition, microglia respond to GABA by suppressing IFN-γ production through inhibition of inflammatory pathways mediated by NF-kB and P38 mitogen-activated protein (MAP) kinases. These inhibitory effects of GABA are partially mimicked by the GABA-A receptor agonist–muscimol and the GABA-B receptor agonist -baclofen, implying functionality for both types of GABA receptors in human microglia (Lee et al., 2011). In mouse retinal microglia, endogenous GABAergic signaling negatively regulated dendritic morphology *in vivo* in the brain as the inhibition of GABA-A or GABA-B receptors resulted in more profound increase in dendritic structures (Fontainhas et al., 2011). A recent characterization in murine primary microglia revealed that (i) microglia exhibit hypermotility upon challenge with *T. gondii*, (ii) secretion of GABA upon *T. gondii* infection, (iii) transcriptional expression of a complete GABAergic machinery including GABA-A receptors, (iv) *T. gondii* infection modulated the expression of the GABAergic machinery, and (v) pharmacological inhibition of GABAergic signaling at different levels (synthesis, receptor, receptor regulator, and VDCC inhibition) abrogated hypermotility of microglia (Bhandage et al., 2019). Jointly, this indicates that *T. gondii* infection activates migration of microglia via GABAergic signaling, similar to DCs.

Additional immune cells that mediate important immune responses to *Toxoplasma* infection, such as monocytes, T cells, NK cells, macrophages, and neutrophils, have been associated with the parasite dissemination, as delineated above. Whether *Toxoplasma* infection modulates GABAergic signaling in other immune cells—if expressed—needs to be addressed in future investigations.

## A Role for Tg14-3-3 in *T. gondii*-Induced Hypermigration of DCs and Microglia

Much of the current work in understanding the *Trojan horse* mechanism has focused on the infected host cell. Cytoskeletal changes with dissolution of podosomes, elevation in motility and transmigration, modulated interactions with extracellular matrix and regulation of integrin receptors, among others, have been reported in DCs or monocytic cells following infection by *T. gondii* (Lambert et al., 2006; Harker et al., 2013; Weidner et al., 2013; Kanatani et al., 2015; Cook et al., 2018; Olafsson et al., 2018).

By analyzing the impact of fractionated total tachyzoite lysates on DC motility, *T. gondii* 14-3-3 (Tg14-3-3), a parasite-derived orthologous protein of the ubiquitously expressed 14-3-3 protein family of eukaryotic cells, was linked to hypermigration of DCs and microglia (Weidner et al., 2016). In the absence of other *T. gondii* proteins, recombinant Tg14-3-3 was sufficient to induce a hypermigratory state in DCs and microglia, with velocities comparable to that of a live *Toxoplasma* infection. Tg14-3-3 was detected in secreted parasite fractions and localized to the parasitophorous vacuolar space in infected DCs. Interestingly, a rapid recruitment of host cell 14-3-3 to the parasitophorous vacuole membrane (PVM) was observed (Weidner et al., 2016). Thus, the PVM may serve as the potential interface for Tg14-3-3 interaction with host cellular proteins.

Little is known about the functions of the 14-3-3 proteins in apicomplexan parasites, and specifically Tg14-3-3 (Assossou et al., 2003, 2004; Lorestani et al., 2012). In mammalian eukaryotic cells 14-3-3 proteins are involved in the MAP kinase-mediated regulation of cellular processes including the organization of the cytoskeleton and the cell motility (Sluchanko and Gusev, 2010). Further, specific human isoforms of 14-3-3 can promote cell migration and metastasis of cancer cells through cytoskeletal remodeling (Somanath and Byzova, 2009; Freeman and Morrison, 2011). It is therefore plausible that Tg14-3-3, directly or indirectly through sequestration of host 14-3-3, impacts on MAP kinase signaling and thereby motility.

## GABAergic Migratory Activation of DCs Can Cooperate With Chemotaxis of *Toxoplasma*-Infected Leukocytes

Upon contact with microbes in peripheral tissues, DC maturation implies changes in the expressed chemokine receptors that license migration to draining lymph nodes. As delineated above, *Toxoplasma*-induced hypermigration of DCs depends on GABAergic signaling (Fuks et al., 2012), but not on classical chemokine receptors, e.g., chemokine receptor 7 (CCR7) or Toll-like receptor (TLR)/MyD88 signaling (Lambert et al., 2006; Olafsson et al., 2018). Yet, hypermigratory DCs down-regulate CCR5 and up-regulate CCR7 upon *Toxoplasma* challenge (Fuks et al., 2012; Weidner et al., 2013), in line with reported chemotactic responses by CD34^+^ DCs (Diana et al., 2004). Secretion of the dense granule protein GRA5 has been associated with the up-regulation of CCR7, and CCR7/CCL19-driven chemotaxis (Persat et al., 2012). Indeed, DCs challenged with soluble GRA5, or live tachyzoites, chemotaxed in a CCL19 gradient (Fuks et al., 2012; Persat et al., 2012; Weidner et al., 2013).

The impact of chemokine receptor modulation in *Toxoplasma*-infected DCs remains however unexplored *in vivo*. *In vitro*, the onset of GABAergic hypermigration following parasite invasion is rapid (minutes) while the onset of measurable chemotactic responses is significantly slower *in vitro* (12–24 h) (Fuks et al., 2012; Weidner et al., 2013; Kanatani et al., 2015). Yet, hypermotility and chemotaxis cooperatively potentiated the speed and directional motility of parasitized DCs *in vitro*. Thus, GABAergic hypermotility and CCR7-mediated chemotaxis may, in theory, jointly potentiate migration of infected DCs *in vivo*, and facilitate parasite dissemination.

## The *Trojan horse* Mechanism and Free Extracellular Tachyzoites: Two Co-Existing Modes of Parasite Dissemination?

Intracellular localization in migratory leukocytes represents a secluded niche for dissemination, partly protecting from immune attack in the hostile extracellular environment. However, despite *T. gondii*'s obligate intracellular existence for replication (Dobrowolski and Sibley, 1996), its extracellular gliding motility mechanism provides a means for migration in the microenvironment in tissues (Barragan and Sibley, 2002). Freshly egressed tachyzoites can actively traverse polarized epithelial and endothelial cell monolayers. The identified process of paracellular transmigration implicates interactions between host cell ICAM-1 and the parasite adhesin MIC2 (Barragan and Sibley, 2002, 2003; Barragan et al., 2005; Furtado et al., 2012b).

Shortly after inoculation of parasites in murine experimental models, leukocyte-associated tachyzoites can be detected in the circulation but also free extracellular tachyzoites that increase in numbers as infection develops (Lambert et al., 2009; Konradt et al., 2016). The relative contribution of these two possible modes for dissemination in natural infections in rodents or humans remains unexplored. However, it is likely that, following systemic dissemination in the blood, passage to the brain occurs with low parasitemia during natural primary infection. Moreover, extracellular tachyzoites are exposed to neutralization by the complement system and IgM (Couper et al., 2005). Also, parasite genotype-related differences have been described. For type II and III strains, the relative leukocyte-associated fraction of tachyzoites predominated early during infection (Lambert et al., 2009), in line with observations of leukocyte-associated type II parasites early after infection (Courret et al., 2006; Unno et al., 2008). In contrast, for type I parasites, the relative extracellular tachyzoite fraction was predominant in the spleen (Lambert et al., 2009). Additionally, transfer of tachyzoites between different leukocyte types has been shown to occur in the blood and tissues (Persson et al., 2007, 2009; Kanatani et al., 2017).

One additional possibility is that infected leukocytes in circulation “deliver” the parasites to the endothelium (Lambert and Barragan, 2010), as recently reported for a pulmonary infection model in mice (Baba et al., 2017). In infection models of toxoplasmosis, both parasitized leukocytes and free extracellular tachyzoites have been suggested to mediate dissemination in ocular infection (Furtado et al., 2012a,b, 2013) and for intraluminal intestinal spreading in mice (Coombes et al., 2013; Gregg et al., 2013). Jointly, this indicates that both intracellular and extracellular dissemination strategies co-exist and may even act in a complementary fashion at different phases of the infection.

## Conclusions and Perspectives

*Toxoplasma gondii* has developed strategies for hijacking the migratory functions of infected leukocytes, which simultaneously serve as a replicative niche. Thus, *T. gondii* reconciles the obligate need for intracellular replication with the establishment of infection in peripheral organs. To this end, *T. gondii* modulates the motogenic GABAergic/VDCC signaling axis of DCs and microglia to hijack migratory functions and promote its dissemination. Similarly, upcoming evidences indicate that parasites, bacteria and viruses modulate GABAergic signaling in immune cells for survival (Fuks et al., 2012; Zhu et al., 2017; Kim et al., 2018).

The impact of GABA and other neuroactive molecules in immune cells is an emerging field. Infection models, such as toxoplasmosis, can increase the current understanding of the GABAergic signaling system in immune cells and on how GABAergic signaling impacts on physiological and pathophysiological conditions. Additionally, novel insights into the pathogenesis of infections are provided. Research from recent years has revealed that immune cells are GABAergic and future studies will likely uncover novel functions for GABA signaling in immune cells.

## Author Contributions

All authors listed have made a substantial, direct and intellectual contribution to the work, and approved it for publication.

### Conflict of Interest Statement

The authors declare that the research was conducted in the absence of any commercial or financial relationships that could be construed as a potential conflict of interest.
